# Macrophage Proteome Analysis at Different Stages of *Mycobacterium avium* Subspecies *paratuberculosis* Infection Reveals a Mechanism of Pathogen Dissemination

**DOI:** 10.3390/proteomes9020020

**Published:** 2021-04-30

**Authors:** Ida L. Phillips, Lia Danelishvili, Luiz E. Bermudez

**Affiliations:** 1 Department of Biomedical Sciences, Carlson College of Veterinary Medicine, Oregon State University, Corvallis, OR 97331, USA; iphillips@tuskegee.edu; 2 Department of Microbiology, College of Sciences, Oregon State University, Corvallis, OR 97331, USA

**Keywords:** proteomics, *Mycobacterium avium* subspecies *paratuberculosis*, macrophage, host response, integrins, spread

## Abstract

Johne’s disease is a chronic and usually fatal enteric infection of ruminants caused by *Mycobacterium avium* subspecies *paratuberculosis* (MAP) and is responsible for hundreds of millions of dollars in losses for the agricultural industry. Natural infection typically begins with bacterial uptake and translocation through the epithelium of the small intestine, followed by ingestion by tissue macrophages and dissemination via the lymphatic or blood system throughout the body. To gain insights into the host responses and adaptation of MAP within phagocytic cells, we utilized the previously developed cell culture passage model, and mass spectrometric-based quantitative proteomic approach. Using the cell culture system, which mimics an in vivo interaction of MAP with intestinal epithelium and tissue macrophages, bacteria were passed through the bovine epithelial cells and, subsequently, used for macrophage infection (termed indirect infection), while uninfected cells and macrophage infection initiated with the culture grown bacteria (termed direct infection) served as controls. Approximately 3900 proteins were identified across all studied groups. The comparison within the subset of proteins that showed synthesis for more than two-fold in the direct infection over the uninfected control revealed an enrichment for the pro-inflammatory pathways such as the NF-κB and cytokine/chemokine signaling, positive regulation of defense response, cell activation involved in the immune response and adaptive immune system. While these responses were absent in the indirect infection, cellular pathways such as cell cycle, healing, regulation of cell adhesion, ensemble of core extracellular matrix proteins, cell surface integrins and proteins mediating the integrin signaling were remarkably high within the indirect infection. In addition to global analysis of the macrophage proteome, we further validated the proteomics data and confirmed that MAP passage through epithelial cells modulates the expression and signaling of integrins in phagocytes. In this study, we demonstrate that predominant expression of integrins in the indirectly infected macrophages allows phagocytic cells to initiate stronger binding and efficient translocation through the endothelial cells, suggesting the important role of integrins in the spread of MAP infection.

## 1. Introduction

*Mycobacterium avium* subspecies *paratuberculosis* (MAP) is a causative agent of Johne’s disease, a severe, chronic and endemic wasting disease that results in the death of ruminant animals [1]. The economic losses of the affected dairy industries in the United States alone have been estimated to be between $200 and $250 million annually due to significant reduction in milk production and increased mortality of animals [2,3,4].

Johne’s disease progression is slow with clinical signs of intermittent diarrhea and weight loss occurring after 2–5 years post-infection. Young ruminants are exposed to MAP while suckling infected dams or through fecal contaminated milk. In utero infection can also occur. The ingested bacteria that reach the small intestine enters the lamina propria through microfold cells and enterocytes [5,6]. The pathogen is then taken up by local tissue macrophages and dendritic cells. Macrophages provide a niche for MAP replication, survival and, later, for spread to the lymph nodes, mammary tissue, liver, and epithelial tissue [7]. The infection and dissemination processes of MAP that occur within the ruminant host are complex, multi-factorial, and occurs in a variety of stages. These factors make it difficult to unravel molecular mechanisms occurring within the host environment at each stage of Johne’s disease. While efforts have been made to establish consistency among large animal models in MAP research [8], experimental discrepancies, duration of the experiment and cost make it difficult to have a precise understanding on cellular mechanisms of MAP pathogenicity.

A novel in vitro cell culture passage model of the intestinal environment and interactions encountered by MAP during different stages of infection has been established based on the current understanding of MAP pathogenesis [9]. This model mimics in vivo infection from the invasion of the intestinal epithelium followed by infection of the host phagocytes and finally returning to the intestinal epithelium during the later stages of infection. In the sequential cell passage model, the initial MAP infection of epithelial mucosal cells does not trigger an inflammatory response, a fact observed in vivo [10]. However, bacteria passage through epithelial cells and then macrophages initiate a pro-inflammatory immune response in the subsequent infection of epithelial cells. Furthermore, these findings are substantiated by demonstrating that MAP genes expressed in the tissue culture cells in vitro are similar to the gene expression levels of bacteria that were recovered from the intestinal mucosa of diseased cattle [9]. The diverse phenotypes of the pathogen reflect the multiple disease stages during the course of MAP infection, and it is likely that varying bacterial phenotypes have significant impact on discrepancies of diagnostic methods and MAP vaccination efficacy [11].

Little is known about MAP dissemination during the course of the infection. The ability of MAP to invade organs like liver, mammary gland and other distant sites has been always difficult to study and analyze. It is a fact that the dissemination of MAP infection not only have consequences for the fitness of the host but also is associated with ability of bacteria to infect other animals. Furthermore, the host factors that contribute to the long incubation period of MAP and stimulate incomplete immune responses that fail to control Johne’s disease progression remain unknown and represent a gap in knowledge. The past work by our group has demonstrated that different MAP phenotypes stimulate diverse cellular responses in the host [9,12]. To gain insights into the host metabolic pathways and immune signaling related to intracellular adaptation and persistence of MAP within phagocytic cells, we employed a mass spectrometric approach and investigated proteome of macrophages that were either directly infected with culture grown MAP (termed as direct infection) or bacteria that were passed through the bovine epithelial cells (termed as indirect infection) and then used for consequent macrophage infection. Our results indicate that the macrophage proteomics of the secondary MAP infection is enriched with proteins that belong to non-inflammatory cellular pathways such as cell cycle, healing, cell surface integrins and SNARE trafficking signaling. Conversely, macrophages of primary or direct infection express the pro-inflammatory response enriched with pathways of microbial killing, immunity activation and cytokine secretion. Identification of specific changes in the macrophage proteome in response to infection with varied phenotypes of MAP significantly expands our understanding on the signaling pathways of the phagocytic cell and reflects the cellular response that may allow the intracellular pathogen to spread and survive within the host for an extended time.

## 2. Methods and Materials

### 2.1. Bacteria

*Mycobacterium avium* subspecies *paratuberculosis* strain K10 (ATCC BAA-968) was cultured at 37 °C for 4–5 weeks on 7H10 Middlebrook agar media supplemented with casein hydrolysate (1 g/L; BD), 10% (*v*/*v*) oleic acid, albumin, dextrose, and catalase (OADC; Hardy Diagnostics; Santa Maria, CA, USA), and ferric mycobactin J (2 mg/L; Allied Monitor, Fayette, MO, USA). Prior to experiments, the bacterial inoculum was made in PBS and passing suspension through a 22-gauge needle 15 times to disperse clumps. After 10 min of settling, top 3/4 of the inoculum was used for experiments as a single-cell suspension as described previously [12].

### 2.2. Mammalian Cell Culture

Madin-Darby bovine kidney (MDBK) epithelial cells (CCL-22), Raw 264.7 macrophage cultures (TIB-71) were obtained from the American Type Culture Collection (ATCC; Manassas, VA, USA). MDBK cells and Raw 264.7 cell lines were cultivated in Dulbecco’s Modified Eagle’s Medium (DMEM) supplemented with 10% heat-inactivated fetal bovine serum (FBS; Gemini Bio-Products; West Sacramento, CA, USA) at 37 °C in 5% CO_2_. Proliferating Bovine Aortic Endothelial Cells (s) were purchased from the Cell Application INC (San Diego, CA, USA). BAOEC were cultured in the Bovine Endothelial Cell Growth Medium (Application INC, San Diego, CA, USA) at 37 °C in 5% CO_2_. The cell viability was determined using the alamarBlue reagent according to the manufacturer’s protocol (ThermoFisher Scientific, Waltham, MA, USA). The cell lines were maintained and stocked according to manufacturer’s protocols.

### 2.3. Invasion and Survival of MAP in RAW 264.7 Macrophages in the Direct Infection Assay

The Raw 264.7 macrophages (10^6^) were seeded in 24-well plates overnight in DMEM and infected with mid-log phase grown MAP at a MOI (multiplicity of infection) of 10. The inoculum was prepared from bacteria grown on 7H10 agar plates as described above. The infection process was synchronized by centrifuging tissue culture plates at 350× *g* for 5 min followed by incubation at 37 °C. After 2 h invasion time, supernatants were removed, and wells were washed three times with Hank’s buffered salt solution (HBSS). In addition, monolayers were treated with amikacin (200 µg/mL) supplemented in the fresh DMEM media for 1 h to remove any remaining extracellular bacteria. Monolayers were lysed with 500 µL of 0.1% Triton X-100 (Sigma-Aldrich, St. Louis, MO, USA) in HBSS at 0 h, 24 h and 120 h, serially diluted and then plated for Colony Forming Unit (CFU) determination on 7H10 agar plates.

### 2.4. Invasion and Survival of MAP in RAW 264.7 Macrophages in the Indirect Infection Assay

The 75 cm^2^ tissue culture flasks were seeded to >80% confluency with MDBK cells and infected with MAP of 7H10 grown plate at MOI of 100. After 2 h, cells were washed three times with HBSS, treated with 200 µg/mL of amikacin for 1 h and replenished with new media. At 24 h and 10 days post-infection of epithelial cells, monolayers were lysed with 0.1% Triton x-100 (Sigma-Aldrich, St. Louis, MO, USA) for 15 min and mechanical disruption with pipetting. Next, the lysates were centrifuged at low speed of 800× *g* for 10 min at 4 °C to remove the macrophage cell debris without pelleting the bacteria. The pellets were discarded and supernatant, containing bacteria with an intracellular phenotype, centrifuged at the higher speed of 10,000× *g* for 20 min. Bacteria were resuspended in HBSS and washed two times in HBSS using centrifugation at 10,000× *g* for 20 min and at 4 °C setting. The recovered bacterial pellet was suspended in RPMI media and used in the indirect infection assay.

**Figure 1:**
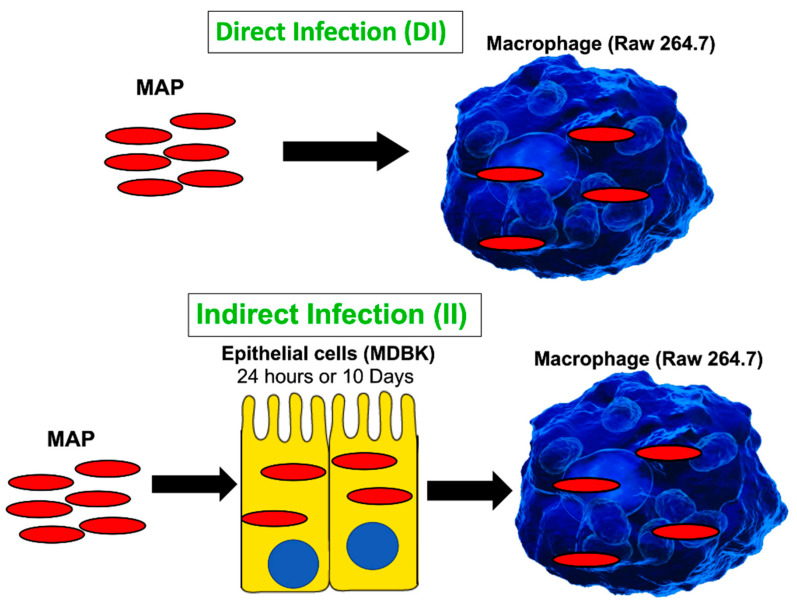
A schematic of in vitro cell culture passage model to mimic the path of MAP infection taking place during crossing the epithelial layer of intestine. The direct infection of macrophages with MAP grown in the culture media serves as a control. The timepoints for the indirect infection include 24 h and 10 days. Samples were collected and processed for quantification of viable bacterial and proteome analysis as described in the materials and methods.

Monolayers of Raw 264.7 cells (10^6^) in 24-well plates were simultaneously infected with passaged MAP of epithelial cells by spinning plates at 350× *g* and as described for the primary infection. The wells were lysed at 0 h, 24 h and 120 h in 1% Triton X-100 (Sigma-Aldrich, St. Louis, MO, USA), serially diluted in HBSS and plated on 7H10 agar enriched with ferric Mycobactin J for quantification of CFU counts of surviving MAP. Figure 1 shows a visual diagram for in vitro assays of the direct and indirect infection.

### 2.5. Protein Sample Preparation and Mass Spectrometric Analysis

The direct and indirect MAP infection assays in Raw 264.7 macrophages were performed as described above with slight modification where 24-well plates were scaled up to the 25 cm^2^ tissue culture flasks. Infected macrophage monolayers from both experimental (infection) and control (uninfected) groups were lysed at 24 h time-point in 3% Sodium dodecyl sulfate (SDS Millipore, Sigma, St. Louis, Mo, USA) supplemented with the protease inhibitor cocktail (ThermoFisher Scientific, Waltham, MA, USA) and via mechanical disruption on a bead-beater. Samples were cleaned using detergent removal kit (ThermoFisher Scientific, Waltham, MA, USA), digested with trypsin at 37 °C overnight and sequenced in the Mass Spectrometry Center at Oregon State University using a Thermo Orbitrap Fusion Lumos MS coupled with a Waters nano-Acquity UPLC system (Waters). All raw files were analyzed using Proteome Discoverer software. After protein identification, we utilized the PANTHER (Protein ANalysis THrough Evolutionary Relationships) and STRING resources to cluster evolutionarily related proteins by molecular function, biological process and by pathways.

### 2.6. The Endothelial Cell Binding Assay

The direct and indirect MAP infection assays in Raw 264.7 macrophages were performed in the 25 cm^2^ tissue culture flasks as described above. After 24 h of infection, cells were fluorescently labeled with DAPI (4′,6-diamidino-2-phenylindole) at 1:5000 dilution for 30 min. The uninfected Raw 264.7 macrophages served as a control for binding assay. Monolayers were detached using 30% mM cold EDTA, counted on a hemocytometer, adjusted to 1 × 10^5^ cells/mL and resuspended in 300 mL of Bovine Aortic Endothelial cell media. The labeled macrophages (direct, indirect or uninfected) were added to confluent monolayers of BAOEC seeded in 24-well plates at 2 × 10^5^ cells/mL and incubated for 2 h. The endothelial cells were washed three times with HBSS and then processed for fluorescent readings using DAPI filter sets on the TECAN infinite 200.

### 2.7. Polarized Endothelial Cell Transwell Assay

The BAOEC endothelial cells (2 × 10^5^/well) were seeded on the 6.5 mm Corning^®^ *Transwell^®^* polycarbonate membrane cell culture inserts with 20 mm pore size (Corning, Tewksbury, MA, USA). The polarized monolayer was achieved after 5 days at 37 °C and 5% CO_2_. The transendothelial resistance was measured using Millicell-ESR apparatus as per manufacturer’s instructions (Millipore). Final values were obtained by subtracting the blank value, and the results were expressed as ohms/cm^2^. The trypan blue dye was used to monitor integrity of the membrane [13]. To establish the translocation rate of Raw 264.7 cells through the endothelial cell monolayer, macrophages of either uninfected or direct and indirect infection groups (prepared as described above) were placed in the upper chamber. After 2 h, transwells were removed and migrated macrophages were stained with Giemsa stain (Sigma-Aldrich, St. Louis, MO, USA) and counted using a brightfield microscope in the lower chamber. The assay was repeated three times.

### 2.8. The Endothelial Cell Binding and Migration Assays via Integrins

For the binding assay, the confluent monolayers of BAOEC were seeded in 24-well plates at 2 × 10^5^ cells/mL. The Raw 264.7 macrophages of uninfected, direct and indirect groups were opsonized with monoclonal anti-mouse anti-CD11b, CD36 or CD 71 antibodies (Santa Cruz Biotechnology, Dallas, TX, USA) for 1 h at a dilution of 1:200, washed with HBSS and stained with DAPI. Macrophages without antibody pre-treatment were used as a control group. Macrophages were incubated with BAOEC monolayers. After 2 h, monolayers were washed three times with HBSS and processed for fluorescent readings.

For the migration assay, the polarized endothelial cells were established as described above. Raw264.7 macrophages of uninfected, direct or indirect infection groups were opsonized with monoclonal anti-mouse CD11b or CD 71 antibodies at a dilution of 1:200 and incubated for 1 h at 37 °C. Cells with no antibody pretreatment served as a control for the integrin migration assay. Macrophages were washed with HBSS and placed on confluent polarized monolayers of endothelial cells. After 2 h, transwells were removed and migrated macrophages were stained with Giemsa stain and counted using a brightfield microscope in the lower chamber. The assay was repeated three times.

### 2.9. Statistical Analysis and Data Interpretation

Statistical significance for binary comparisons was performed on four independent experiments of proteomics data using the Student’s *t* test. For the binding assay, results are reported as the mean of at least two independent experiments ± standard error, where comparisons between experimental groups and control groups were determined using the Student’s *t* test with *p* < 0.05 denoting statistical significance. Survival curve data were analyzed using Kaplan–Meier Survival Analysis. GraphPad Prism version 8.0 software was used for the construction of graphs, data interpretation, and statistical analysis.

## 3. Results

### 3.1. The Cell Passage Model for Studying a Mechanism of MAP Persistence and Dissemination

To readily understand the progression of infection in the bovine host without using a prohibitive and not practical bovine model, our group has developed an in vitro cell culture system that mimics the interactions between the MAP pathogen and the host intestine over the course of infection [9]. The goal of this study was to identify the cellular processes stimulated by MAP, after crossing the intestinal epithelial barrier, and to understand a molecular mechanism that promoted bacterial persistence and, later, spread within the host. Figure 1 schematically shows the experimental design where bacteria enter the MDBK epithelial cells (representing enterocytes of the host intestine) followed by bacteria leaving the epithelium and, subsequently, ingested by macrophages (Figure 1A) where MAP replicate and disseminate throughout the body. The direct infection of macrophages (Figure 1B) is used as a control to identify how host responses differ between infections of the laboratory cultured bacteria and intracellular MAP of epithelial cell phenotype. Furthermore, because it is unknown how long MAP remains within enterocytes before the pathogen is ingested by macrophages, we studied intracellular phenotypes of MAP at 24 h and 10 days post-infection. Twenty-four-hour infection of epithelial cells represent a model of bacterial transverse to the lamina propria in short time, while the 10-day infection corresponds to long-term incubation. Interestingly, epithelial cells at 24 h and 10 days post-infection remained visually intact and healthy. The epithelial cell viability was checked every day using the alamarBlue viability assay and no cytotoxicity was observed over 10-day MAP infection. The intracellular bacteria persisted within cells and did not exit to the supernatant. The intracellular MAP was recovered at both timepoints and used for macrophage infection to determine differences between these groups, if any.

### 3.2. MAP Passage through Epithelial Cells Increases Bacterial Invasion and Survival in Phagocytic Cells

**Figure 2:**
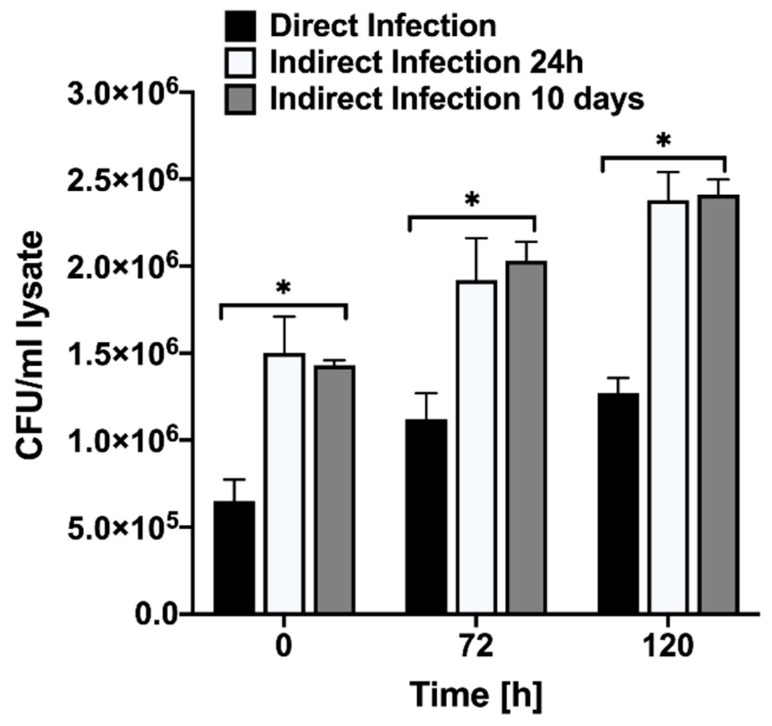
MAP growth dynamics in Raw 264.7 cells. The macrophage monolayers were infected with MOI of 10 bacteria obtained either from the 7H10 culture media at mid-log growth phase or recovered from the passage through MDBK epithelial cells. The invasion rates at 0 h and intracellular growth kinetics over 4 days of infection were monitored by plating the serial dilutions of viable bacterial on 7H10 agar plates and recording the colony forming units. Data represent the means ± standard deviations (SD) obtained from three independent experiments performed in duplicates. * *p* < 0.05 statistical significance between the indirect infection of both time points and the direct infection.

In the model of the late phase of intestinal mucosal reinfection, the previous study has been demonstrated that MAP recovered from the macrophage infection stimulates very strong pro-inflammatory phenotype in epithelial cells. These findings directly corelate with inflammatory process within animal intestine before bacteria is shed into the environment [9]. In attempts to gain insights into early stages of MAP pathogenesis mechanisms, in the current study, we investigated the cellular changes that are triggered by bacteria after gaining access into the host through gastrointestinal tract and crossing the epithelial layer. The intracellular MAP was recovered after passaging through epithelial cells at 24 h and 10-day post-infection and these phenotypes were tested for the pathogen’s ability to survive in phagocytic cells. The time killing dynamics clearly demonstrate significant differences in invasion and survival of MAP during direct and indirect infection of macrophages (Figure 2). The uptake percentage of indirect infection was much higher than direct infection. While MAP of both phenotypes survived in macrophages, over the course of the infection there was a significant increase in passaged bacterial survival when compared to non-passage infection. These results indicate that MAP passage through the epithelium alters bacterial phenotype in a way that increases better recognition and uptake of the pathogen by macrophages and, later, intracellular survival as well.

### 3.3. Macrophage Proteome of Indirect/Passaged MAP Significantly Differs from the Direct Infection

**Figure 3:**
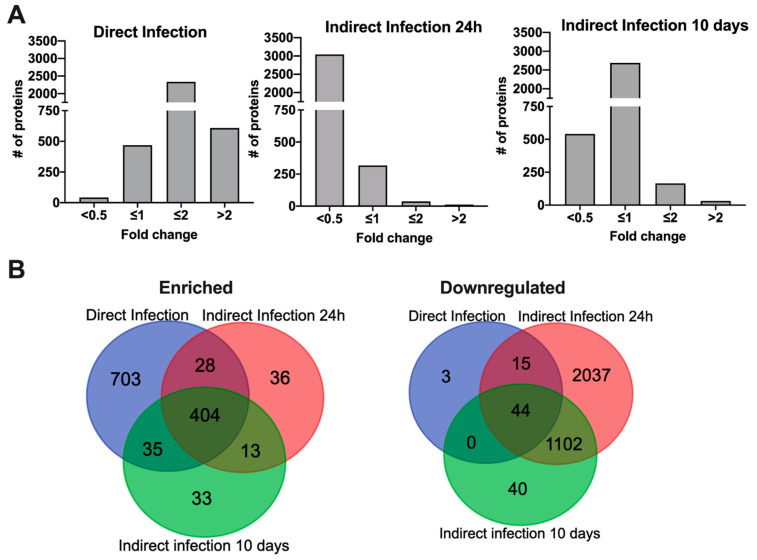
Differentially synthesized macrophage proteome during infection of MAP with varied phenotypes. (**A**) Histograms show the distributions of fold changes of proteins enriched in direct and indirect infections of 24 h and 10 days passaged MAP; (**B**) Venn Diagrams display proteins that are induced or repressed with ≥ 2-fold when compared to the uninfected control and are statically significant.

After crossing the epithelial layer of intestine, MAP is subsequently uptaken by tissue macrophages. To gain insights into the host cell metabolic and regulatory pathways promoting an intracellular phenotype and adaptation of MAP within phagocytic cells, we employed a mass spectrometric-based quantitative proteomics approach. The pathogen was passed through bovine epithelial cells for 24 h and 10 days and then infected Raw 264.7 macrophages (indirect infection). Uninfected cells and MAP infection utilizing laboratory cultured bacteria (direct infection) served controls for macrophage proteome analysis. Approximately 3900 proteins were identified across the uninfected, direct and indirect infection groups. The mass spectrometry proteomics data have been deposited to the ProteomeXchange Consortium [14] via the PRIDE [15] partner repository with the dataset identifier PXD024539. Histograms of Figure 3A demonstrate the distribution of the average fold changes for host proteins of experimental groups versus uninfected control. The comparison within the subset of proteins that showed synthesis for more than two-fold identified 404 shared and uniquely synthesized 703, 36 and 33 proteins in the direct and indirect infections of 24 h and 10 days passaged bacteria, respectively (Figure 3B). Proteome analysis of downregulated proteins revealed only 62 host proteins in the direct infection including 44 proteins shared between all studied groups (Figure 3B). Interestingly, the synthesis of significant number of host proteins were inhibited during indirect infection of both time-points (1142 and 3139 proteins, respectively at 24 h and 10 days).

**Figure 4:**
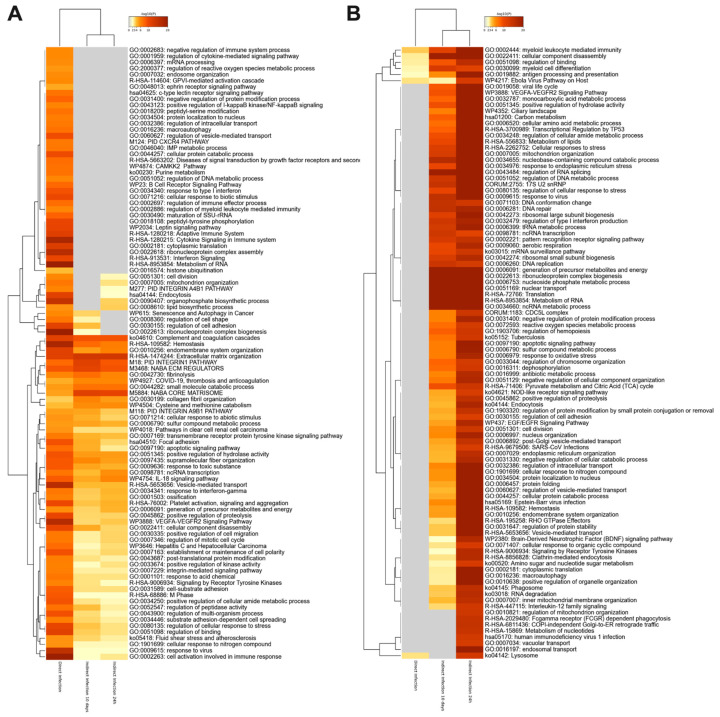
The pathway enrichment analysis. The gene enrichment and pathway analysis were performed for host proteins exhibiting significant changes in (**A**) synthesis or (**B**) downregulation during MAP infection of macrophages. The data were evaluated using the Metascape program and enriched terms with the best *p*-values are displayed in a dendrogram. The significant terms were also hierarchically clustered into a tree based on Kappa-statistical similarities among their gene memberships. While the heatmap cells are colored by their *p*-values, the grey cells indicate the lack of enrichment for that term in the corresponding gene list.

The heat map analysis of the gene ontology (GO) shown in Figure 4 displays the KEGG pathway enrichment for molecular interaction, reaction and relation networks. We found that these systems significantly differed between direct and indirect MAP infections, while majority of activated or suppressed pathways were similar within the indirect infection group of 24 h and 10 days. The predominant functional groups enriched in the direct infection were related to cell activation involved in the immune response and adaptive immune system such as cytokine-mediated signaling, cytokine production, response to type I interferons, cellular response to lipopolysaccharide, positive regulation of NF-kB kinase signaling, regulation of inflammatory response, cytosolic DNA-sensing pathway, and positive regulation of defense response (Figure 4A). The cellular signaling pathways activated in the indirect infection groups included positive regulation of cell migration, integrin-mediated signaling pathways, regulation of binding and NABA extracellular matrix, IL-18 signaling known to be involved in host defense, inflammation, and tissue regeneration. The overall analysis of suppressed proteome of the indirect infection of both 24 h and 10 days displays inhibition of RNA metabolism, translation and protein folding processes, and confirms less inflammatory phenotype in phagocytic cells when compared to the direct infection (Figure 4B). This observation may suggest that extended dwelling in the enterocyte or epithelial cell has a positive impact on the ability of MAP to remain undetected by the host immune system.

Due to the fact that the SNAREs, endosomal and lysosomal proteins are implicated in various intracellular trafficking steps, playing a role in the mycobacterial phagosome maturation process and fusion with late endosomes [16,17,18], we describe this group of proteins in Table 1. In addition, Table 2 details the list of integrin proteins known to regulate the adhesive activity of the cell and notably synthesized during the indirect infection of both timepoints.

**Figure 5:**
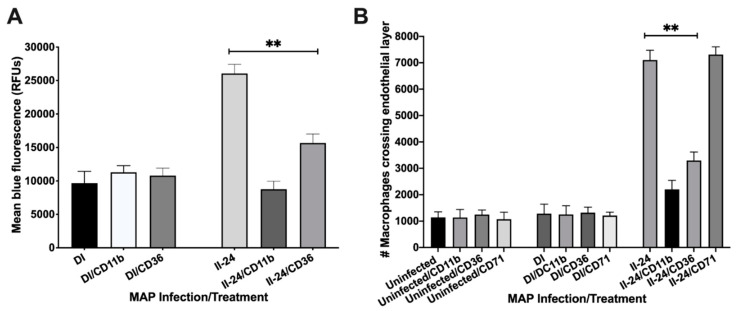
The endothelial layer binding and translocation ability of MAP infected macrophages. (**A**) The DAPI-labeled Raw 264.7 macrophages of direct and indirect groups (10^5^ cells/mL) were preincubated with antibodies (or not) and then added to confluent monolayers of BAOEC for 2 h. Endothelial monolayers were washed three times with HBSS and then processed for fluorescent readings; (**B**) The polarized endothelial cell layer was formed on transwell membranes as described in the materials and methods. After confirmation the integrity of endothelium with the trypan blue dye, macrophages of either uninfected or direct and indirect infection groups with and without antibody preincubation were placed in the upper chamber. After 2 h, migrated macrophages were stained with Giemsa stain and counted using a brightfield microscope. Data represent the means ± standard deviations (SD) obtained from three independent experiments. **, *p* < 0.01 between binding as well as crossing capacity of macrophages of the indirect infection with antibody treatment of CD11b and CD36 versus the same group without specific antibody treatment.

The macrophage enrichment with integrin proteins in the indirect infection allows phagocytes to bind to and cross the endothelial cell layer more efficiently. To determine if integrin proteins stimulated better binding and transport of MAP infected macrophages through endothelial layer, we: (1) fluorescently labeled macrophages of direct and indirect infection of 24 h and assessed the binding capacity to endothelial monolayer established in the tissue culture plates, and (2) utilized the transwell system and evaluated the translocation of infected phagocytic cells of different phenotypes through polarized endothelium layer. Our results demonstrate that the exposure of macrophages of the indirect infection with the endothelial cell monolayer displayed significantly higher/firm binding ability to BAOEC cells when compared to the fluorescence levels recorded for macrophages of the direct infection (Figure 5A). We also found that number of macrophages crossing the polarized endothelium were significantly greater in the 24 h indirect infection group versus the direct infection measured by counting the Giemsa-stained cells translocated in the lower chamber of the transwell (Figure 5B). Furthermore, to establish the direct association of integrins with a stronger attachment to the endothelium and possible role in movement to distance location throughout the body, we used two integrin specific anti-CD11b and anti-CD36 monoclonal antibodies and non-integrin related the cell surface transferrin receptor protein 1 (CD71) antibody as a control. The CD71 receptor was also found enriched in macrophages of the indirect infection. The phagocytic cells of 24 h indirect infection was opsonized to block integrin functions and then retested for binding and translocation ability through endothelial layer. While CD11b and CD36 pretreated groups of the 24 h indirect infection significantly blocked the macrophage binding to the epithelial cell layer and influenced crossing ability, opsonization of macrophages of the direct infection or no infection did not display any changes in the attachment or binding capability of phagocytes (Figure 5A,B). The anti-CD71 treatment had no detected activity in preventing bind and/or crossing property of macrophages via endothelial cells.

## 4. Discussion

Tissue macrophages and dendritic cells play a crucial role during the establishment, maintenance and progression of Johne’s disease [19,20]. The proper activation of innate immune mechanisms by these specialized phagocytic cells is important for MAP infection clearance; however, MAP is a successful pathogen capable of manipulating and triggering inadequate cellular responses, which in turn largely contribute to long term and silent incubation period of the pathogen in the host. To understand the molecular mechanism of MAP prolonged survival within the ruminant host requires the capacity to study the infection pathology in a controlled research environment. A defined model of Johne’s disease which accurately depicts the host immune responses and underlying changes caused by MAP infection, is not available. While in vivo methods have been developed to study MAP pathogenesis [21], drawbacks include the necessity of large research facilities, extended time of disease progression, and high overhead costs. The experimental outcome and reproducibility can be influenced and affected by the selected breeds, age of inoculation, including MAP strain, route and dosage used during infection [21]. Alternatively, researchers have turned to the use of rodents to study the ability of MAP to invade the intestinal mucosa and disseminate to peripheral tissues [5] and to identify and validate vaccine candidates [5,22]. Mouse models, however, offer limited insight on the complete disease progression because they do not exhibit intestinal inflammation in the same manner that ruminants do in the advanced stages of Johne’s disease.

In recent years, more elaborate in vitro systems, mimicking the different environments encountered by MAP during infection of the host, have been developed [9,23,24,25]. The in vitro passage models first described in our laboratory represent a novel technique in which the intricate host-microbe interactions during the various stage of infection may be analyzed in a simplified manner. The multicellular model, used in this study, replicates the path that MAP takes from its uptake by the intestinal epithelium, spreading to the tissue phagocytes, and ultimate returning to the intestinal epithelium during the later stages of infection [9]. There is a solid evidence in literature demonstrating that bacterial exposure to varied environmental conditions can promote different bacterial phenotypes, influencing its fitness within the host. Our hypothesis is that MAP translocation via the lamina propria and passage through the epithelium sharpens the pathogen’s ability to efficiently survive the next phase of infection, while causing a minimum immune reaction and damage to the host cells. We believe that MAP “intracellular phenotype” developed within epithelial cells alters the pathogen recognition by immune system and contribute to bacterial persistence in phagocytes. Therefore, macrophages constitute a nidus of infection until the later stages of the disease, when MAP would leave macrophages to gain access back to the intestinal lumen and, ultimately, out of the host environment.

There are many fundamental aspects that remain unknown during MAP infection of host phagocytic cells. Without exclusively focusing on single pathway and establishing enrichment for individual proteins using traditional immunoassay methods, we utilized the quantitative MS/MS analysis and obtained global proteome print that provides insight into many cellular signaling mechanisms activated during MAP infection. To determine how crossing the epithelial cell layer (as it occurs during MAP infection of intestinal barrier) changes the host cellular responses promoting MAP persistence phenotype in macrophages and contribute to the long-term survival in the host, we used the culture passage model and quantitatively analyzed proteome of macrophages. Our results demonstrate that direct MAP infection of macrophages mediates the pro-inflammatory response activating the NF-κB and cytokine/chemokine signaling, positive regulation of defense, cell activation involved in the immune response and adaptive immune system, reactive oxygen species, metabolic processes, and macroautophagy. The protein enrichment analysis of the indirect MAP infection of both 24 h and 10 days timepoints reveals the absence of similar inflammatory activity. In the indirect infected macrophages, we observed of stimulation of cellular pathways such as cell cycle, healing, regulation of cell adhesion, ensemble of core extracellular matrix proteins including ECM glycoproteins, collagens and proteoglycans. In addition, the considerably higher number of cell surface integrins and proteins belonging to the integrin-mediated signaling were identified in the indirect infection when compared to direct infection.

The synthesis of SNARE (soluble N-ethylmaleimide-sensitive factor attachment protein receptors) trafficking proteins were largely present in both the direct and indirect infection experimental groups. The enrichment of SNAREs clearly demonstrates the macrophage attempt to clear infection via innate defense mechanisms in both groups. However, it has been demonstrated that MAP, similar to other successful mycobacterial pathogens, has evolved virulence mechanisms to prevent the host killing mechanisms [26,27,28]. The SNAREs are endosomal and lysosomal proteins responsible for membrane fusion, movement of organelles throughout the cells and are vital in bacterial and viral infections, intracellular transport and release [29,30,31]. One of the identified SNARE trafficking proteins, Rab32, was highly synthesized by macrophages during the indirect infection. Rab32 is member of a large family of small GTPase that control membrane identity, vesicle formation, motility, and fusion [32]. Rab32 is associated with vesicle remodeling and plays an important role in mediating antimicrobial activity by promoting phagosome maturation at an early phase of infection [33]. The differential recruitment of Rab32 can control the recruitment of cathepsin D to the phagosome, altering the phagolysosome biogenesis. Cathepsin D was also found to be highly synthesized in the indirect infections of both timepoints. While upregulation of both host protein during bacterial infection leads to elimination of the pathogen by lysosomal degradation, the pathogens including *M. tuberculosis* and *M. avium* has acquired strategies to block the function or trafficking process of these target proteins to the phagosome and, thus, allowing intracellular growth and persistence [34]. The Vamp8 protein, enriched in the indirect infection of 24 h, is a well-studied SNARE protein. Its trafficking from lysosomes to phagosomes can mediate the fusion process with endosomes or lysosomes [17]. Interestingly, it has been demonstrated that *Leishmania* can avoid intracellular killing by the innate immune defenses by cleaving Vamp8 with a secreted effector protease enzyme resulting and preventing the NOX2 recruitment to the phagosome [35]. In addition, *Salmonella typhimurium*-generated phosphatidylinositol 3-phosphate promotes the recruitment of Vamp8 to the bacterial invasion site aiding the pathogen uptake [36]. The Vamp8 has been shown to selectively traffic to *Coxiella burnetii* containing vacuoles, suggesting an important role in the pathogenesis of this intracellular pathogen [37].

Furthermore, our results indicate that MAP passage through epithelial cells increased synthesis of integrins in macrophages, while this expression was mainly downregulated in the direct infection cohort. The effect of MAP on integrin expression in immune cells has not been reported in the literature. Given the prominent role of integrins in migration, adhesion to vascular endothelium, and interactions with T cells, it is important to consider the potential role of integrins in the pathogenesis of MAP infection. We hypothesized that the upregulation of integrins in macrophages of passaged MAP infection would initiate stronger binding to the endothelial cells and transport through the endothelium more efficiently, later, helping bacterial spread and dissemination. The proteome research provides a refreshing source for interrogating complex disease processes and gaining insights. However, it is also important to pair proteomic findings with biological assays and validate findings. Therefore, we selected CD11b and CD36 targets to establish direct relationship between high levels of integrin synthesis in the indirect infected macrophages and increased binding and translocation of this group of phagocytes through endothelium.

The integrin ITGAM encodes the CD11b chain of the Mac-1 integrin (αMβ2; CD11b/CD18; complement receptor-3). It also regulates macrophage polarization and is critical for adhesive interactions of monocytes and macrophages [38]. In addition, CD11b is responsible for the process of immune cell attachment to endothelial cells and migration within the extracellular matrix, and blocking its function using anti-CD11 antibodies inhibits phagocytic adhesion and transmigration through endothelial cells [39]. Our results demonstrate that while expression of CD11b integrin was markedly downregulated in the direct MAP infection, the synthesis of this integrin in the indirect infections of 24 h and 10 days was 4- and 3-fold higher, respectively, when compared with the uninfected control. CD36 is a member of scavenger receptor B family, and transmembrane glycoprotein receptor that is expressed in a variety of cells, including adipocytes, endothelium, epithelium, myocytes, platelets cells, monocytes, and macrophages [40]. Recent studies have also revealed that macrophage CD36 functions as a signaling molecule, transmitting signals via specific Src and MAP kinases, including Lyn and JNK1 and JNK2 upon binding to ligands, including oxidized low density lipoprotein [41]. These signals are required for oxLDL internalization and foam cell formation [40]. CD36 is involved in several physiological and pathological processes, including immunity, lipid absorption, storage, metabolism, and inflammation [42].

In this study, the binding and endothelial cell transwell migration assays directly validate proteomics data that MAP passage through epithelial cells alters the way in which bacteria are able to modulate expression of the host cell integrins. We show that macrophages of the indirect infection were capable to bind and migrate through a polarized layer of endothelium cells at significantly higher rates when compared with macrophages of the direct infection. By blocking the function of selected integrins using the anti-CD11b and CD36 antibodies, we observe the decreased ability of macrophages of the indirect infection to adhere and cross endothelial cells.

The proteomic data obtained in this study provide a new perspective in Johne’s disease pathogenesis and suggest that the phenotypic remodeling of MAP within the epithelial cells remarkably changes the way the host phagocytic cells respond to MAP infection, allowing the prolonged survival of bacteria. The ability of MAP to stimulate synthesis of integrins in macrophages, permitting efficient attachment and translocation through the endothelial layer, also reveals the cellular responses that may allow the intracellular pathogen to disseminate throughout the host.

## Figures and Tables

**Table 1:** The endosome-lysosome trafficking-related proteins identified in the proteome of MAP-infected macrophages. Direct infection (DI), Indirect Infection of 24 h passaged MAP (II-24), and Indirect Infection of 10 days passaged MAP (II-10).

Proteins	Infection Group	Function
Vamp8	II-24	Regulates autophagosome–lysosome fusion
Vamp3	DI, II-24	Involved in the vesicular transport from the late endosomes to the trans-Golgi network
Vps18	II-24, II-10	Protein trafficking, formation of early endosomes, late endosomes, and lysosomes
Lamtor1	II-24	Lysosomal motility, activation on the late endosome as well as endosomal biogenesis
Rab32	II-24	Regulators of membrane trafficking pathways in eukaryotic cells; Endosome-mediated membrane trafficking
Rab3iL1	II-24	Regulates synaptic vesicle exocytosis
Hpse	II-10	Endoglycosidase that cleaves heparan sulfate proteoglycans
Glb1, Hexb, Gm2a	II-10	Cleaves beta-linked terminal galactosyl residues from gangliosides
Cathepsin D	II -24, II-10	Acid protease intracellular protein breakdown activity
Arsb	II-10	Lysosomal transport, autophagy, degranulation of neutrophils
CD63	II-10	Intracellular vesicular transport processes
SypL1	II-10	Regulates exocytosis, small cytoplasmic transport vesicles
Vps37c	DI	Required for the sorting of ubiquitinated transmembrane proteins into internal vesicles of multivesicular bodies
Litaf	DI	Lipopolysaccharide-induced tumor necrosis factor-alpha factor. Plays a role in endosomal protein trafficking and in targeting proteins for lysosomal degradation
SLCL5a3	DI	Lysosome peptide/histidine transporter
Ar18a	DI	Plays a role in lysosome motility

**Table 2:** The list of integrins highly synthesized by macrophages during MAP infection.

Proteins	Infection Group	Function
Itga5	DI, II-24, II-10	Composed of an alpha subunit and a beta subunit that function in cell surface adhesion and signaling
Adam8	DI (Inhibited in II-24 and II 10)	A disintegrin and metalloprotease domain implicated in a variety of biological processes involving cell–cell and cell–matrix interactions
Fibulin1	II-24, II-10	ECM protein that stabilizes collagen and other ECM proteins
Fibronectin/Fn1	DI, II-24, II-10	Cell adhesion and migration processes including embryogenesis, wound healing, blood coagulation, host defense, and metastasis
CCL4	DI, II-24, II-10	Secreted effector with chemokinetic and inflammatory functions
CD36	II-24, II-10	Involved in a variety of adhesive processes
Itgam/CD11b	II -24, II-10 (Inhibited in DI)	Adherence of neutrophils and monocytes to endothelium; involved in the phagocytosis of complement coated particles

## Data Availability

The mass spectrometry proteomics data is available at the ProteomeXchange Consortium under the dataset identifier PXD024539.

## References

[1] National Research Council (2003). Diagnosis and Control of Johne’s Disease.

[2] Barratt A.S., Arnoult M.H., Ahmadi B.V., Rich K.M., Gunn G.J., Stott A.W. (2018). A framework for estimating society’s economic welfare following the introduction of an animal disease: The case of Johne’s disease. PLoS ONE.

[3] Carter M.A. (2012). Prevalence and prevention of paratuberculosis in North America. Jpn J. Vet. Res..

[4] Kovich D.A., Wells S.J., Friendshuh K. (2006). Evaluation of the Voluntary Johne’s Disease Herd Status Program as a source of replacement cattle. J. Dairy Sci..

[5] Bermudez L.E., Petrofsky M., Sommer S., Barletta R.G. (2010). Peyer’s patch-deficient mice demonstrate that Mycobacterium avium subsp. paratuberculosis translocates across the mucosal barrier via both M cells and enterocytes but has inefficient dissemination. Infect. Immun..

[6] Pott J., Basler T., Duerr C.U., Rohde M., Goethe R., Hornef M.W. (2009). Internalization-dependent recognition of Mycobacterium avium ssp. paratuberculosis by intestinal epithelial cells. Cell Microbiol..

[7] Antognoli M.C., Garry F.B., Hirst H.L., Lombard J.E., Dennis M.M., Gould D.H., Salman M.D. (2008). Characterization of Mycobacterium avium subspecies paratuberculosis disseminated infection in dairy cattle and its association with antemortem test results. Vet. Microbiol..

[8] Hines M.E., Stabel J.R., Sweeney R.W., Griffin F., Talaat A.M., Bakker D., Benedictus G., Davis W.C., de Lisle G.W., Gardner I.A. (2007). Experimental challenge models for Johne’s disease: A review and proposed international guidelines. Vet. Microbiol..

[9] Everman J.L., Eckstein T.M., Roussey J., Coussens P., Bannantine J.P., Bermudez L.E. (2015). Characterization of the inflammatory phenotype of Mycobacterium avium subspecies paratuberculosis using a novel cell culture passage model. Microbiology.

[10] Koets A.P., Eda S., Sreevatsan S. (2015). The within host dynamics of Mycobacterium avium ssp. paratuberculosis infection in cattle: Where time and place matter. Vet. Res..

[11] McBride J.W., Walker D.H. (2010). Progress and obstacles in vaccine development for the ehrlichioses. Expert Rev. Vaccines.

[12] Patel D., Danelishvili L., Yamazaki Y., Alonso M., Paustian M.L., Bannantine J.P., Meunier-Goddik L., Bermudez L.E. (2006). The ability of Mycobacterium avium subsp. paratuberculosis to enter bovine epithelial cells is influenced by preexposure to a hyperosmolar environment and intracellular passage in bovine mammary epithelial cells. Infect. Immun..

[13] Mukherjee T., Squillantea E., Gillespieb M., Shao J. (2004). Transepithelial electrical resistance is not a reliable measurement of the Caco-2 monolayer integrity in Transwell. Drug Deliv..

[14] Deutsch E.W., Bandeira N., Sharma V., Perez-Riverol Y., Carver J.J., Kundu D.J., Garcia-Seisdedos D., Jarnuczak A.F., Hewapathirana S., Pullman B.S. (2020). The ProteomeXchange consortium in 2020: Enabling ‘big data‘ approaches in proteomics. Nucleic Acids Res..

[15] Perez-Riverol Y., Csordas A., Bai J., Bernal-Llinares M., Hewapathirana S., Kundu D.J., Inuganti A., Griss J., Mayer G., Eisenacher M. (2019). The PRIDE database and related tools and resources in 2019: Improving support for quantification data. Nucleic Acids Res..

[16] Wesolowski J., Paumet F. (2010). SNARE motif: A common motif used by pathogens to manipulate membrane fusion. Virulence.

[17] Dingjan I., Linders P.T.A., Verboogen D.R.J., Revelo N.H., Ter Beest M., van den Bogaart G. (2018). Endosomal and Phagosomal SNAREs. Physiol. Rev..

[18] Fratti R.A., Chua J., Deretic V. (2002). Cellubrevin alterations and Mycobacterium tuberculosis phagosome maturation arrest. J. Biol. Chem..

[19] Weiss D.J., Evanson O.A., Moritz A., Deng M.Q., Abrahamsen M.S. (2002). Differential responses of bovine macrophages to Mycobacterium avium subsp. paratuberculosis and Mycobacterium avium subsp. avium. Infect. Immun..

[20] Rathnaiah G., Zinniel D.K., Bannantine J.P., Stabel J.R., Grohn Y.T., Collins M.T., Barletta R.G. (2017). Pathogenesis, Molecular Genetics, and Genomics of Mycobacterium avium subsp. paratuberculosis, the Etiologic Agent of Johne’s Disease. Front. Vet. Sci..

[21] Begg D.J., Whittington R.J. (2008). Experimental animal infection models for Johne’s disease, an infectious enteropathy caused by Mycobacterium avium subsp. paratuberculosis. Vet. J..

[22] Bannantine J.P., Everman J.L., Rose S.J., Babrak L., Katani R., Barletta R.G., Talaat A.M., Grohn Y.T., Chang Y.F., Kapur V. (2014). Evaluation of eight live attenuated vaccine candidates for protection against challenge with virulent Mycobacterium avium subspecies paratuberculosis in mice. Front. Cell. Infect. Microbiol..

[23] Marfell B.J., O’Brien R., Griffin J.F. (2013). Global gene expression profiling of monocyte-derived macrophages from red deer (Cervus elaphus) genotypically resistant or susceptible to Mycobacterium avium subspecies paratuberculosis infection. Dev. Comp. Immunol..

[24] Casey M.E., Meade K.G., Nalpas N.C., Taraktsoglou M., Browne J.A., Killick K.E., Park S.D., Gormley E., Hokamp K., Magee D.A. (2015). Analysis of the Bovine Monocyte-Derived Macrophage Response to Mycobacterium avium Subspecies Paratuberculosis Infection Using RNA-seq. Front. Immunol..

[25] McLoughlin K.E., Nalpas N.C., Rue-Albrecht K., Browne J.A., Magee D.A., Killick K.E., Park S.D., Hokamp K., Meade K.G., O’Farrelly C. (2014). RNA-seq Transcriptional Profiling of Peripheral Blood Leukocytes from Cattle Infected with Mycobacterium bovis. Front. Immunol..

[26] Arsenault R.J., Li Y., Bell K., Doig K., Potter A., Griebel P.J., Kusalik A., Napper S. (2012). Mycobacterium avium subsp. paratuberculosis inhibits gamma interferon-induced signaling in bovine monocytes: Insights into the cellular mechanisms of Johne’s disease. Infect. Immun..

[27] Tessema M.Z., Koets A.P., Rutten V.P., Gruys E. (2001). How does Mycobacterium avium subsp. paratuberculosis resist intracellular degradation?. Vet. Q..

[28] Arsenault R.J., Maattanen P., Daigle J., Potter A., Griebel P., Napper S. (2014). From mouth to macrophage: Mechanisms of innate immune subversion by Mycobacterium avium subsp. paratuberculosis. Vet. Res..

[29] Garg H., Joshi A. (2012). SNAREs in HIV-1 assembly. Commun. Integr. Biol.

[30] Becken U., Jeschke A., Veltman K., Haas A. (2010). Cell-free fusion of bacteria-containing phagosomes with endocytic compartments. Proc. Natl. Acad. Sci. USA.

[31] Lee H.J., Woo Y., Hahn T.W., Jung Y.M., Jung Y.J. (2020). Formation and Maturation of the Phagosome: A Key Mechanism in Innate Immunity against Intracellular Bacterial Infection. Microorganisms.

[32] Stenmark H. (2009). Rab GTPases as coordinators of vesicle traffic. Nat. Rev. Mol. Cell Biol..

[33] Hu Z.Q., Rao C.L., Tang M.L., Zhang Y., Lu X.X., Chen J.G., Mao C., Deng L., Li Q., Mao X.H. (2019). Rab32 GTPase, as a direct target of miR-30b/c, controls the intracellular survival of Burkholderia pseudomallei by regulating phagosome maturation. PLoS Pathog..

[34] Danelishvili L., Bermudez L.E. (2015). Mycobacterium avium MAV_2941 mimics phosphoinositol-3-kinase to interfere with macrophage phagosome maturation. Microbes Infect..

[35] Podinovskaia M., Descoteaux A. (2015). Leishmania and the macrophage: A multifaceted interaction. Future Microbiol..

[36] Dai S., Zhang Y., Weimbs T., Yaffe M.B., Zhou D. (2007). Bacteria-generated PtdIns(3)P recruits VAMP8 to facilitate phagocytosis. Traffic.

[37] Campoy E.M., Mansilla M.E., Colombo M.I. (2013). Endocytic SNAREs are involved in optimal Coxiella burnetii vacuole development. Cell Microbiol..

[38] Podolnikova N.P., Kushchayeva Y.S., Wu Y., Faust J., Ugarova T.P. (2016). The Role of Integrins alphaMbeta2 (Mac-1, CD11b/CD18) and alphaDbeta2 (CD11d/CD18) in Macrophage Fusion. Am. J. Pathol..

[39] Zhang Q.Q., Hu X.W., Liu Y.L., Ye Z.J., Gui Y.H., Zhou D.L., Qi C.L., He X.D., Wang H., Wang L.J. (2015). CD11b deficiency suppresses intestinal tumor growth by reducing myeloid cell recruitment. Sci. Rep..

[40] Park Y.M., Febbraio M., Silverstein R.L. (2009). CD36 modulates migration of mouse and human macrophages in response to oxidized LDL and may contribute to macrophage trapping in the arterial intima. J. Clin. Invest..

[41] Guy E., Kuchibhotla S., Silverstein R., Febbraio M. (2007). Continued inhibition of atherosclerotic lesion development in long term Western diet fed CD36o /apoEo mice. Atherosclerosis.

[42] Silverstein R.L., Febbraio M. (2009). CD36, a scavenger receptor involved in immunity, metabolism, angiogenesis, and behavior. Sci. Signal..

